# Stability of daily rectal movement and effectiveness of replanning protocols for sparing rectal doses based on the daily CT images during proton treatment for prostate cancer

**DOI:** 10.1002/acm2.13015

**Published:** 2020-09-05

**Authors:** Yoshikazu Maeda, Yoshitaka Sato, Kazutaka Yamamoto, Hiroyasu Tamamura, Makoto Sasaki, Nobukazu Fuwa, Shigeyuki Takamatsu, Kyo Kume

**Affiliations:** ^1^ Proton Therapy Center Fukui Prefectural Hospital Fukui Fukui Japan; ^2^ Department of Radiotherapy Ise Red Cross Hospital Ise Mie Japan; ^3^ Department of Radiation Therapy Kanazawa University Hospital Kanazawa Ishikawa Japan; ^4^ Research & Development Department The Wakasa Wan Energy Research Center Tsuruga Fukui Japan

**Keywords:** adaptive radiotherapy, image‐guided radiotherapy, in‐room CT image guidance, prostate cancer, proton therapy

## Abstract

**Purpose:**

To evaluate the optimal period of replanning to spare the rectal dose by investigating daily rectal movements during computed tomography (CT) image‐guided proton therapy for prostate cancer.

**Materials and methods:**

To evaluate the optimum reference period for replanning, we analyzed 1483 sets of daily CT (dCT) images acquired from 40 prostate cancer patients and measured the daily rectal movement along the anterior–posterior direction based on the simulator CT (sCT) images and dCT images. We calculated daily dose distributions based on initial plans on the sCT images and replans on the dCT images for 13 representative patients, and evaluated daily dose volume histograms (DVHs) for the prostate, seminal vesicles, and rectum.

**Results:**

The rectal anterior side on the dCT images around the seminal vesicles largely deviated toward the anterior side relative to the position on the reference sCT images, but the deviation decreased by referring to the dCT images and became nearly zero when we referred to the dCT images after 10‐day treatment. The daily DVH values for the prostate showed good dose coverage. For six patients showing rectal movement toward the anterior side, the daily rectal DVH (V_77%_) showed a 3.0 ± 1.7 cc excess from the initial plan and this excess was correlated with 9.9 ± 6.8 mm rectal movement. To identify the patients (37.5% in total) for whom the replanning on the 10th‐day and 20th‐day CT images reduced the V_77%_ excess to 0.4 ± 1.5 cc and −0.2 ± 1.3 cc, respectively, we evaluated the accumulated mean doses with a 1.2 cc criterion.

**Conclusion:**

Our data demonstrate that the daily movement of the rectal anterior side tends to move toward the anterior side, which results in a rectal overdose, and the mean of the movement gradually decreases with the passage of days. In such cases, replanning with the reference CT after 10 days is effective to spare the rectal dose.

## 1 INTRODUCTION

Proton therapy is now regarded as a common application for prostate cancer, and the numbers of patients undergoing proton therapy have continued to increase due to this therapy's benefit of the sharp distal dose fall‐off beyond the Bragg Peak, which spares organs at risk (OARs). The use of proton therapy is thus expected to reduce the rates of gastrointestinal (GI) and genitourinary (GU) acute and late toxicities compared to the dose distribution provided by conventional photon radiotherapy.^1^ In Japan, patients with prostate cancer undergo fractionated proton radiotherapy with a prescribed dose of 74–78 Gy in 37–39 fractions and 70 Gy in 28 fractions as unified protocols. Clinical trials have been conducted to test the effectiveness of hypofractionated proton therapy using a delivered dose of 63 Gy in 21 fractions with the goal of increasing the treatment throughput and achieving higher tumor control. Since the treatment period still lasts 4–8 weeks, highly precise image guidance for the daily placement of radiation with respect to the target is a key technique to take advantage of the physical selectivity of proton therapy. This has motivated particle therapy vendors to provide 3D volumetric image guidance as a standard function, enabled by cone beam computed tomography (CBCT) or in‐room CT imaging in the current therapy facilities.^2^ With these setups, in principle, one can identify the target and OAR locations on daily CT images and validate the effect of daily changes of patient anatomies on the dose distributions. This could not be done with the two‐dimensional (2D) conventional kV x‐ray image guidance used in most particle therapy facilities in the past.

With the use of opposed lateral beams as is now done widely for proton prostate cancer therapy, the movements of pelvic organs along the beam axis matter for the target dose coverage, and the movements along directions lateral to the beam axis matter for the dose to OARs and thus for the GI and GU acute and late toxicities. The stability of the pelvic organs' daily positions and the stability of the shape of the anterior rectal wall placed at nearby targets over the superior–inferior (SI) direction are important factors in the control of the daily dose to the rectum. In this context, we previously investigated the effects of daily organ motion on prostate treatment for ten patients by using daily CT images acquired throughout the patients' in‐room CT image‐guided proton treatment.^3^ The results demonstrated that daily movements of the anterior rectal wall along the anterior–posterior (AP) direction from the referenced simulator CT images tended to increase from the inferior side to the superior side, due to the daily rectal deformation. The resulting averaged positional deviation was 5 mm at the inferior side and 15 mm at the superior side around the seminal vesicles (SVs). Thus, if we take these averaged positional deviations as a margin for the creation of the planning organ at risk volume (PRV) of the rectum, the resulting rectal dose value does not meet the dose constraint, for example, V_60Gy_ < 18%, which is used as the one of our rectal dose constrains^4^ and was reported originally by Nagoya city university.^5^

In principle, uniform irradiation to the target volume with the same dose formation as that used in the original planning is not sufficient to maintain the daily dose under the planning conditions for a deformable OAR, for example, the rectum. One of the best approaches to this issue is the use of online adaptive planning in combination with CT image guidance, and simulation studies using daily CT images acquired during the treatment have thus been conducted.^6^, ^7^, ^8^ However, further research is needed to examine the contouring and quality assurance of online planning, and this must be done for clinical practice. A replanning protocol based on the statistical knowledge of daily anatomical movements over treatment periods may provide much simpler and more realistic clinical applications compared to the online adaptation of radiation formation to daily random changes. To address this concern, a method of plan selection based on library data sets was proposed for dealing with the daily positional changes of patients' pelvic anatomies.^9^

In the present study, we performed novel analyses to evaluate the replanning protocol for rectal dose sparing based on daily CT images acquired for image guidance prior to proton beam irradiation. In our clinical experience with proton prostate treatment using in‐room CT image guidance, the daily positions of the prostate and the anterior rectal wall identified on online daily CT (dCT) images tended to show differences from the positions in the simulator CT (sCT) images acquired at approx. 10 days pretreatment. We created a replan by referring to the new CT images for particular patients if the dose validation showed its necessity.

We thus studied the stability of the daily movements of the anterior side of the rectal wall by referring to dCT images with four times higher values compared to our previous findings.^3^ In this work, first we evaluated the mean, the random errors, and the systematic errors of daily movement of the rectal anterior wall along the SI direction based on the same analyses method as previous work.^3^ As new approach in this evaluation, the dCT images acquired from the first treatment day to the 20th treatment day were used sequentially as the reference images in addition to sCT images, and then we evaluated the optimal period of replanning by using dCT images as a reference. Second, we examined the impact of these movements on the daily proton dose, and a clinical application was simulated in order to evaluate the effectiveness of the replanning protocol on daily dose parameters.

## 2 MATERIALS AND METHODS

### 2.A Patient data, immobilization, and CT image acquisition

This study was approved by the Institutional Review Boards of our hospitals. We analyzed the dCT images and sCT images that had been acquired for 40 patients undergoing prostate cancer treatment.^4^ All of the patients gave written informed consent to participate in this study. Among them, 27 patients diagnosed with low‐risk prostate cancer were treated with a delivered dose of 74 Gy in 37 fractions, and the other 13 patients diagnosed with intermediate‐to‐high‐risk cancer were treated with 78 Gy in 39 fractions. For all patients, the patient positioning was done using in‐room CT‐image guidance without the use of implanted prostate fiducial markers or a rectal balloon. As described previously,^4^ all patients were scanned in the supine position with a suction‐type fixed bag (RSF‐19Gl; Engineering System Co., Nagano, Japan). The sCT images and MRI images were acquired approx. 10 days before the patient's first treatment. The dCT images were acquired by a self‐propelled CT scanner on rails (Aquilion LB, Canon Medical Systems, Tochigi, Japan) installed in one of the gantry rooms at the Fukui Prefectural Hospital Proton Therapy Center. The daily volume of the bladder was monitored by ultrasound scans to ensure that it was similar to the volume of the sCT images, and patients drank water when the bladder was not sufficiently distended. The defecation and exhaust gases are managed in order to maintain the condition of the rectum as that observed on the sCT images. The dCT images were acquired with a tube current of 150 mA and potential of 120 kV, while the sCT scans were acquired with a current of 480 mA, and reconstructed with a slice thickness of 2 mm and a transversal pixel size of 1.07 × 1.07 mm^2^, which were the same conditions as for the sCT scans.

### 2.B Data processing and image registration procedure

We analyzed a total of 1483 sets of CT images of the 40 patients. We created regions of interest (ROIs) for the prostate, SVs, and rectum on the sCT image and dCT images. First the ROIs of the sCT images were created by experienced radiation oncologists with the support of MRI images. Second the dCT images were registered into the sCT images by referring bony structure using a rigid image registration (MIM Maestro ver. 6.8, MIM Software, Cleveland, OH, USA), and then all ROIs were produced on the dCT images performing a deformed image registration of ROIs of the sCT images using MIM Software. Finally radiation oncologists and an experienced medical physicist carried out the manual correction and validation of ROIs on all sets of dCT images.

Then, to evaluate daily movements of the patients' anatomy in respect to the reference CT images, all dCT images with ROIs were registered to the reference CT images by simulating prostate‐rectum boundary (PRB) registration as the same procedure described previously^3^: First, each of the dCT images was registered to the reference CT image with respect to the bony structure by using the linear matching algorithm included in the MIM Maestro software program, with which we selected the region of pelvic bones in CT images and carried out the registration in a translation along three directions and a rotation around three orthogonal axes. Then, all of the daily ROIs were transferred to the reference CT images in the same coordinate system. To evaluate further translational corrections for PRB registration along the SI directions and the AP directions, the geometric center from the daily coordinates of the prostate ROI to the center of the reference ROI was matched first; we then matched the daily posterior edge of the prostate facing the rectum to the reference with a translational correction along the AP direction by means of the least squares method, in which the corrections were determined by the formula $\sum_{i}\left(y_{i}^{d}‐y_{i}^{r}\right)/N$, where $y_{i}^{d}$ and $y_{i}^{r}$ are the coordinates along the AP direction of the prostate from the dCT images and the reference, respectively, and N denotes the number of points constituting the posterior edge. We noted that the same registration procedure was also carried out for the prostate cancer treatment in our proton facility using the in‐room CT image‐guided system in which the boundary between the posterior sides of the prostate and the rectum on the dCT images was manually registered to the one on the reference images with visual image guidance after bony registration, and we compared the translational corrections used in the treatment with the corrections calculated by the ROIs basis, and the standard deviations of the difference was 1.0 and 1.3 mm along the SI and AP directions, respectively.^4^ In contrast to the approach used in the previous study,^3^ we sequentially referred the sCT images and the dCT images acquired between the first day and the 20th day of the patient's treatment for the reference CT images in order to evaluate the optimum reference period for replanning. All procedures described above and those performed later with the reference CT images were repeated sequentially.

### 2.C Evaluation of the interfractional movement for the anterior side of the rectum

**Fig. 1:**
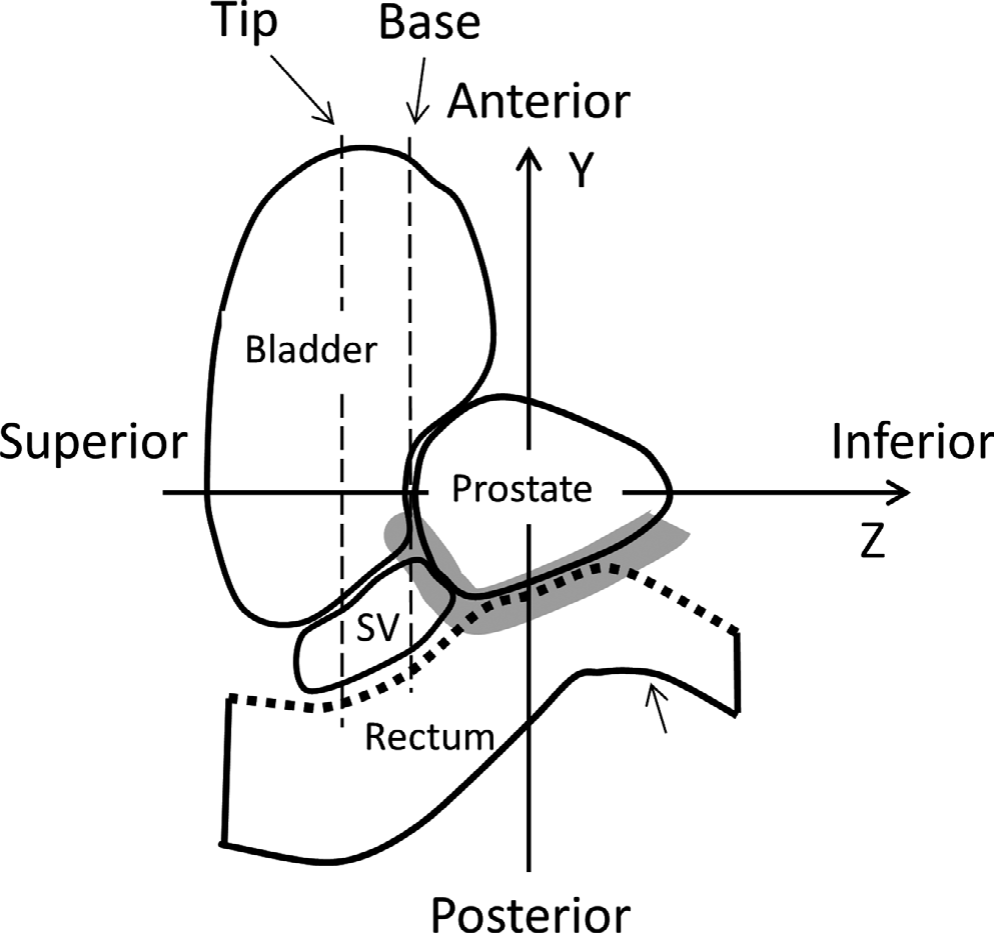
Schematic view of pelvic anatomy on the sagittal plane, and the coordinates. *Dotted line:* The anterior side of the rectum used for the daily movement evaluation. The *gray hatched lines* indicate the contour lines of the prostate used for the prostate‐rectum boundary (PRB) registration.

Figure 1 provides a schematic view of the pelvic anatomy over the sagittal plane and the coordinates that were used. Orthogonal axes were defined based on the center of the prostate of the reference. The daily positional movement of the anterior side of the rectum along the AP (Y) direction was evaluated with respect to the reference over the SI (Z) direction. The SI position was defined as a function of the scaled Z coordinates in order to align each position of the rectal wall in the SI direction with the averaged size of the prostate relative to the individual size for all patients.

The average size of the prostate among all 40 patients was 36.9 ± 7.7 [mm], and thus the prostate was ±18.5 [mm] in the scaled Z coordinate. In addition, the position of the base and tip portion of the SV shown in Fig. 1 were also indicated at Z =  −18.5 [mm] and −26.2 [mm], respectively, where the base and tip were defined at the Z position of the superior prostate and at a distance from the base equivalent to 2/3 of the length of the SVs, respectively. The averaged distance between the tip and the base position was 7.7 ± 5.0 [mm].

### 2.D Mean, random errors, and systematic errors

We evaluated the mean, the random errors, and the systematic errors of the daily positional deviations of the anterior side of the rectum as a function of the scaled Z coordinate with respect to the reference. In the following analysis, as noted before, the reference images were sequentially scanned from the sCT images to the dCT images acquired from the first treatment day to the 20th treatment day, denoted by r = 0, 1–20, respectively. For each patient denoted by *i*, the mean value ($m_{i}^{r}$) and standard deviation ($\sigma_{i}^{r}$) were evaluated as a function of the scaled Z coordinate from the measurements among responsible fractions by $m_{i}^{r}=\sum_{d=r+1}^{n}\left(P_{d}‐P_{r}\right)/N$ and $\sigma_{i}^{r}=\sqrt{\sum_{d=r+1}^{n}{\left(P_{d}‐P_{r}‐m_{i}^{r}\right)}^{2}/N}$, where $P_{d,r}$ are the daily and referenced positions of the anterior rectum along the AP direction, respectively, N denotes the total number of responsible fractions, and n denotes the total fractions. The mean value ($m^{r}$), random error ($\sigma^{r}$), and systematic error ($\Sigma^{r}$) were obtained by the mean of $m_{i}^{r}$, the root mean square of $\sigma_{i}^{r}$, and the standard deviation of $m_{i}^{r}$, respectively, among all patients. We also estimated the average positional deviations using the formula 2.5Σ + 0.7σ proposed by Van Herk et al. to provide the margin values with 95% confidence.^10^ We also obtained the mean value, random error, and systematic error of the positional correction of the prostate along the AP and SI directions with respect to the reference number as described above to see how these values changed relative to the reference number.

### 2.E Planning simulation and daily proton dose calculation

The initial treatment plan and replans were created using a proton treatment planning system (PTPS) (XiO^®^‐N; Elekta Corp., Stockholm, Sweden) based on a passive scattering method used in our proton therapy facility (Hitachi, Tokyo). The planning procedure and parameter settings were essentially the same as those used for the planning in our current prostate cancer treatment as described previously.^4^ Briefly stated, a clinical target volume (CTV) was created by experienced radiation oncologists. The CTV for low‐risk prostate cancer consists of the prostate only or the prostate and a part of the proximal area of the SVs, whereas most of the SVs were included additionally for the patients with higher risk cancer. A planning target volume (PTV) was created by expanding the CTV by 6 mm with the exception of the use of a 5‐mm margin exclusively at the posterior side of the CTV. The beam isocenter was set to the geometrical center of the PTV. The rectal volume for the DVH evaluation along the slice direction was determined by the size of the CTV with the expansion of 10 mm along the SI direction, and the averaged rectal volume used for the DVH evaluation was 41.4 ± 9.1 cc. The representative rectal dose constraint of V_60Gy_ < 18% was maintained by tuning a collimator margin to the PTV around the posterior side, and the averaged V_60Gy_ in cubic centimeter was 7.3 ± 2.5 cc. We aimed to maintain V_95%_ = 100% for cases in which the CTV is expanded by 3 mm, where V_D_ represents the relative volume receiving at least a specified absolute or relative dose, D. However most of the planning particularly for higher risk cancer did not meet V_95%_ = 100% for the expanded CTV as well as the CTV to keep the rectal dose condition due to the inclusion of the SVs in the CTV. In this case, we gave priority to maintaining the dose coverage of V_95%_ = 100% for the prostate and the rectal dose condition of V_60Gy_ < 18% while we deteriorated the dose coverage of the SVs. The replanning with the dCT images was created using the same procedure as used initially for the sCT images.

Based on the above parameters determined in the initial plan and replans, we performed daily dose calculations using all of the dCT images registered on the reference images with respect to the bony structure by applying the daily isocenter corrections along the AP and SI directions evaluated by the PRB procedure, and a delivered dose on each of the dCT images was set to be 2 Gy. The detail workflow for the daily dose calculations was described in our previous work.^4^ Unlike in our previous work, we used dose calculation software (Axion4S ver. 1.0; Hyogo Ion Beam Medical Support, Hyogo, Japan) into which the material parameters, physics parameters, beam data, and CT translation tables used in our PTPS were imported. A pencil beam algorithm that was similar to the algorithm in our PTPS was used. The Axion4S software can work via the DICOM interface with MIM maestro software in a common platform, and thus the dose calculations and analyses can be performed more efficiently compared to the PTPS. The difference in the dose distribution calculated by the Axion4S software and that calculated by our PTPS was validated in several phantoms made of water, bone, and lung equivalent materials (Kyoto Kagaku Co., Ltd., Kyoto, Japan) as well as with the CT images used for the prostate cancer treatment. Overall agreement was achieved between the two calculations, but the distance of the lateral penumbra in the Axion4S software was observed to be approx. 0.5 mm smaller than the distance in our PTPS system; this resulted in values that were a few percent different in the dose volume histogram (DVH) of the rectum from the DVH of the PTPS. The dose distributions of the initial plans as well as those of the replans were therefore recalculated in the Axion4S software and used for later analyses.

The daily DVHs for the prostate and rectum were calculated for 2 Gy irradiation, and the daily changes in the value of V_95%_ were evaluated for the prostate and the SVs; the daily changes in the value of V_77%_ in cubic centimeter were evaluated for the rectum. For the rectal V_77%_ evaluation, the region of the rectal volume on the dCT images along the slice direction was maintained to the one on the sCT images. Seventy‐seven percent of the total delivered dose of 78 Gy corresponds to 60 Gy. For the SVs, we evaluated the daily value of V_95%_ using full SVs volume also for the lower risk cancer to see their deviation. We evaluated the mean, standard deviation, and range of daily DVH values of each organ for each patient over the entire treatment period.

In order to make primary decisions for the replanning according to the daily dose parameters, we also calculated the accumulated mean dose values using the following formula $\sum_{i=0}^{N_{m}}V_{i}/N_{m}$, where *i* denotes the treatment day running from the first day up to the monitoring day, *V_i_* denotes the daily dose parameters, and *N_m_* denotes the total number of accumulated days. We evaluated the overdose criteria for the rectum for the accumulated mean value to identify problematic patients during the early period of the treatment. We also evaluated the correlation between daily rectal movement and rectal dose parameters to provide the empirical function for the dose estimation based on the online measurement of the daily rectal movement.

## 3 RESULTS

Table 1 summarizes the mean values ($m^{r}$), systematic errors ($\Sigma^{r}$), and random errors ($\sigma^{r}$) of the translational corrections by the PRB registration along the AP (Y) and SI (Z) directions with respect to the bone registration for the 40 patients by referring to the sCT images and dCT images acquired on the 1st, 5th, and 10th days of treatment. The means and systematic errors are the largest when the sCT images are referenced. This indicates that the position of the prostate with respect to the bony structure at ~10 days before the treatment differs from the position during the treatment and tended to be at the anterior side.

**Table 1:** The mean values ($m^{r}$), systematic errors ($\Sigma^{r}$), and random errors ($\sigma^{r}$) of translational corrections by the PRB registration along the AP (Y) and SI (Z) directions with respect to the bone registration for the 40 patients.

Ref. CT	AP, Y (mm)	SI, Z (mm)
	$m^{r}$($\Sigma^{r}$, $\sigma^{r}$)	$m^{r}$($\Sigma^{r}$, $\sigma^{r}$)
sCT	−1.2 (2.0,1.9)	1.2 (2.0,1.4)
1st dCT	−0.1 (1.2,1.8)	0.4 (0.9,1.4)
5th dCT	0.0 (1.1,1.7)	0.0 (0.9,1.4)
10th dCT	0.0 (1.0,1.6)	−0.2 (0.8,1.3)

sCT: the simulator CT images referenced; ith: daily CT image (dCT) acquired at the ith day during the treatment.

By referring to dCT images, the mean of the corrections showed a smaller deviation among the patients compared to the means obtained with the sCT images, and these values show a decreasing tendency as the reference day increases.

**Fig. 2:**
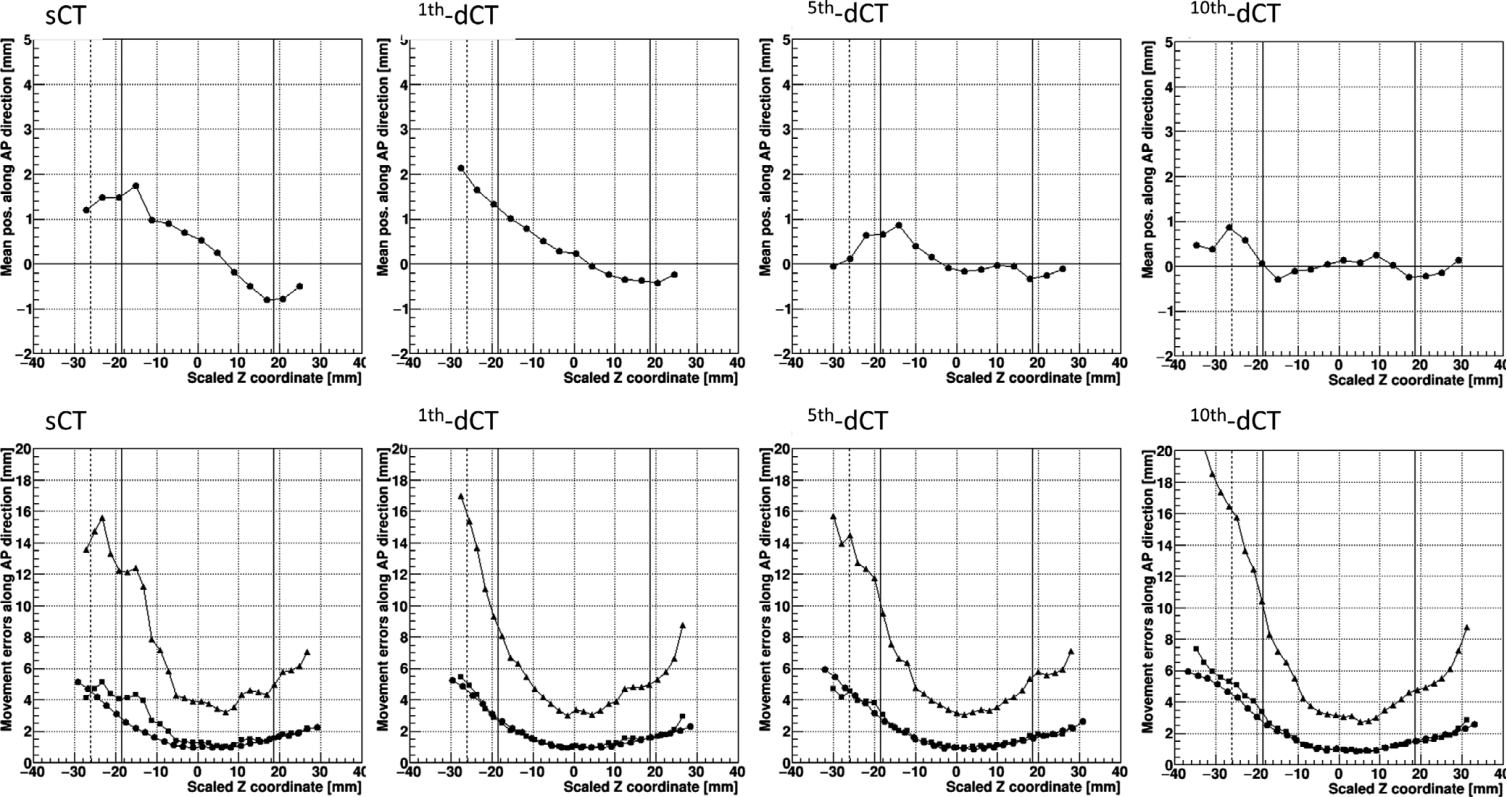
The means ($m^{r}$, *circles, upper panels*), systematic errors ($\Sigma^{r}$, *squares, lower panels*), random errors ($\sigma^{r}$, *circles, lower panels*) and average positional deviations (*triangles, lower panels*) along the AP direction for the anterior wall of the rectum. *Vertical solid lines* show the mean position of the superior and inferior prostate, respectively. *Vertical dotted line:* The mean position of the SV tip. Figures in the first, second, third, and fourth columns indicate the means and errors obtained by referring to the simulator CT images (sCT, r = 0) and the dCT images acquired on the 1st (r = 1), 5th, and 10^th^ days, respectively.

Figure 2 illustrates the respective means, systematic errors, random errors, and average positional deviations obtained along the AP direction for the anterior wall of the rectum by referring to the sCT images and dCT images acquired on the 1st, 5th, and 10th days of treatment. The positive and negative Z directions correspond to the inferior and superior sides, respectively, as shown in Fig. 1.

In general for all references, the errors and average positional deviations of the rectal wall tended to increase from the inferior side to the superior side, and they showed a maximum value around the region of the SVs. The values of these errors and the averaged positional deviations obtained by referring to the sCT images as a reference for the 40 patients in this study showed qualitative agreement with published results for 10 patients.^3^ Our new finding is that the means of the rectal movement around the SV region largely deviated toward the anterior side from the reference sCT images. The positive deviation of the means and the amount of systematic errors obtained with the use of the dCT images as the reference showed a decreasing tendency as we referred the dCT images acquired at the treatment day passed. The mean deviation almost coincided with the baseline values (within 1 mm) when the daily CT images acquired on the 10th day were used in the PRB registration.

**Fig. 3:**
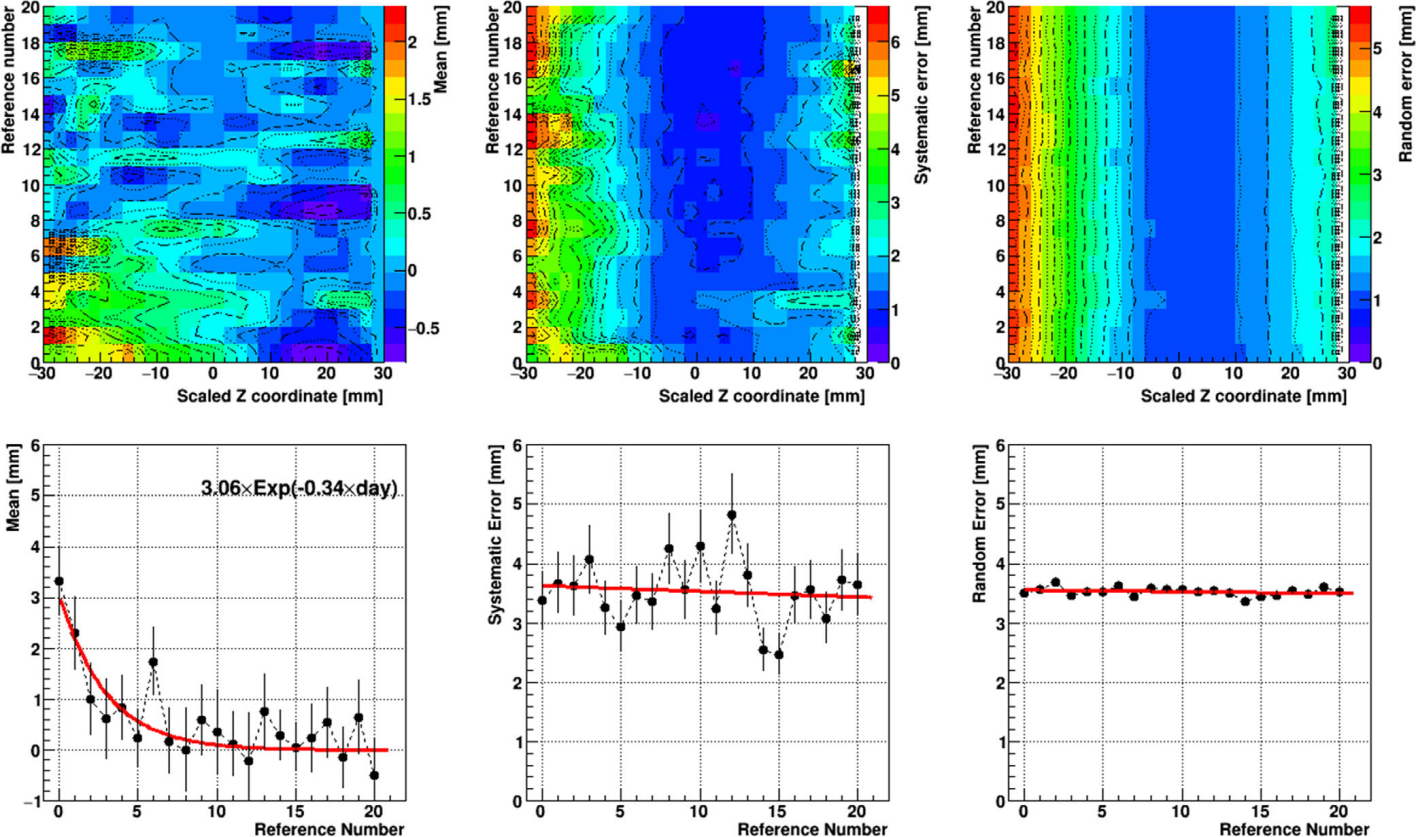
The means ($m^{r}$ *, left panels*), systematic errors ($\Sigma^{r}$ *, middle panels*), and random errors ($\sigma^{r}$, *right panels*) for the anterior wall of the rectum. In the *upper panels*, the vertical and horizontal axes indicate the reference number of CT images and the scaled Z position with respect to the prostate center, respectively. The lower panels show the means and errors over the reference number for the anterior wall of the rectum around the SVs at the SI position of Z = −18.5 to −26.2 mm. *Solid lines* indicate the fit with an exponential and linear function for the means, systematic errors, and random errors, respectively.

The upper panels in Fig. 3 show the means, systematic errors, and random errors of the anterior rectal wall that we obtained by continuously changing reference images from the sCT images to the dCT images acquired between the first day and the 20th day. As demonstrated in Fig. 2, the means of the deviation from the reference in particular around the superior side close to the position of the SVs decreased as the reference number increased; the errors showed a similar tendency but their changes were not significant compared to those of the means.

In particular, the means, systematic errors, and random errors of the anterior rectal wall around the SVs at the SI position of Z =  −18.5 to −26.2 mm are shown in Fig. 3 (lower panels) and were recalculated for the patients whose daily rectal wall around the SVs was observed to move toward the anterior side compared to the position in the sCT images. The mean value decreased as the reference number increased, and the dependence on the reference number was fitted by a χ^2^ method using an exponential function with the slope parameter of −0.34. The systematic and random errors showed a weak decreasing tendency as the reference number increased. These results revealed that the daily shape of the anterior rectal wall tends to coincide better with the shape on the reference images when we refer to the dCT images acquired after 10 days of treatment have passed.

**Fig. 4:**
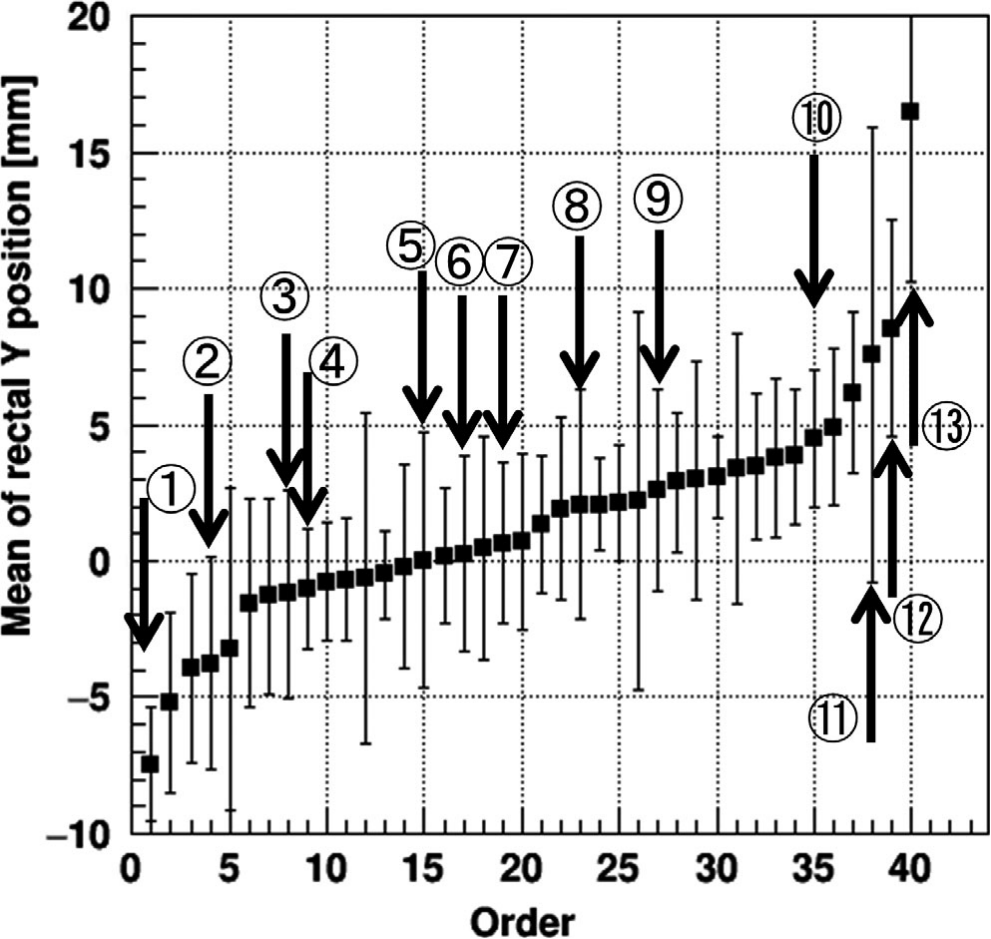
Means (*squares*) with the random errors (*bars*) for the anterior wall of the rectum at Z =  −18.5 to −26.2 mm around the SV region for each patient with reference to the simulator CT images. The horizontal axis of each figure shows the order number of patients according to the size of the mean value of the daily movement. *Arrows* and *numbers* indicate the patient numbers used for the daily dose evaluations.

Figure 4 illustrates the means of daily movement for the anterior wall of the rectum around the SVs at the SI positions of Z =  −18.5 to −26.2 mm for each patient with reference to the sCT images as a function of the order number according to the size of the mean values. A patient with a negative mean value indicates that the anterior rectal wall moves toward the posterior side with respect to the rectal position in the sCT images, whereas a positive value indicates movement toward the anterior side with respect to the reference. These data demonstrate that for approx. 60% of the total patients in the PRB registration, the anterior wall of the rectum around the SV region during the treatment moves toward the anterior side.

For the evaluation of the daily dose parameters, we selected 13 patients based on the size of their rectal movement (see Fig. 4) and categorized them into three groups: (a) Patients #1–#4 showed negative movement of the anterior rectal wall; (b) Patients #5–#7 showed mean values of the movement close to the baseline; and (c) Patients #8–#13 showed positive movement. Patients #1, #5, #11, and #12 underwent the lower risk prostate cancer treatment, and Patients #2‐#4, #6‐#10, and #13 underwent the higher risk prostate cancer treatment.

**Fig. 5:**
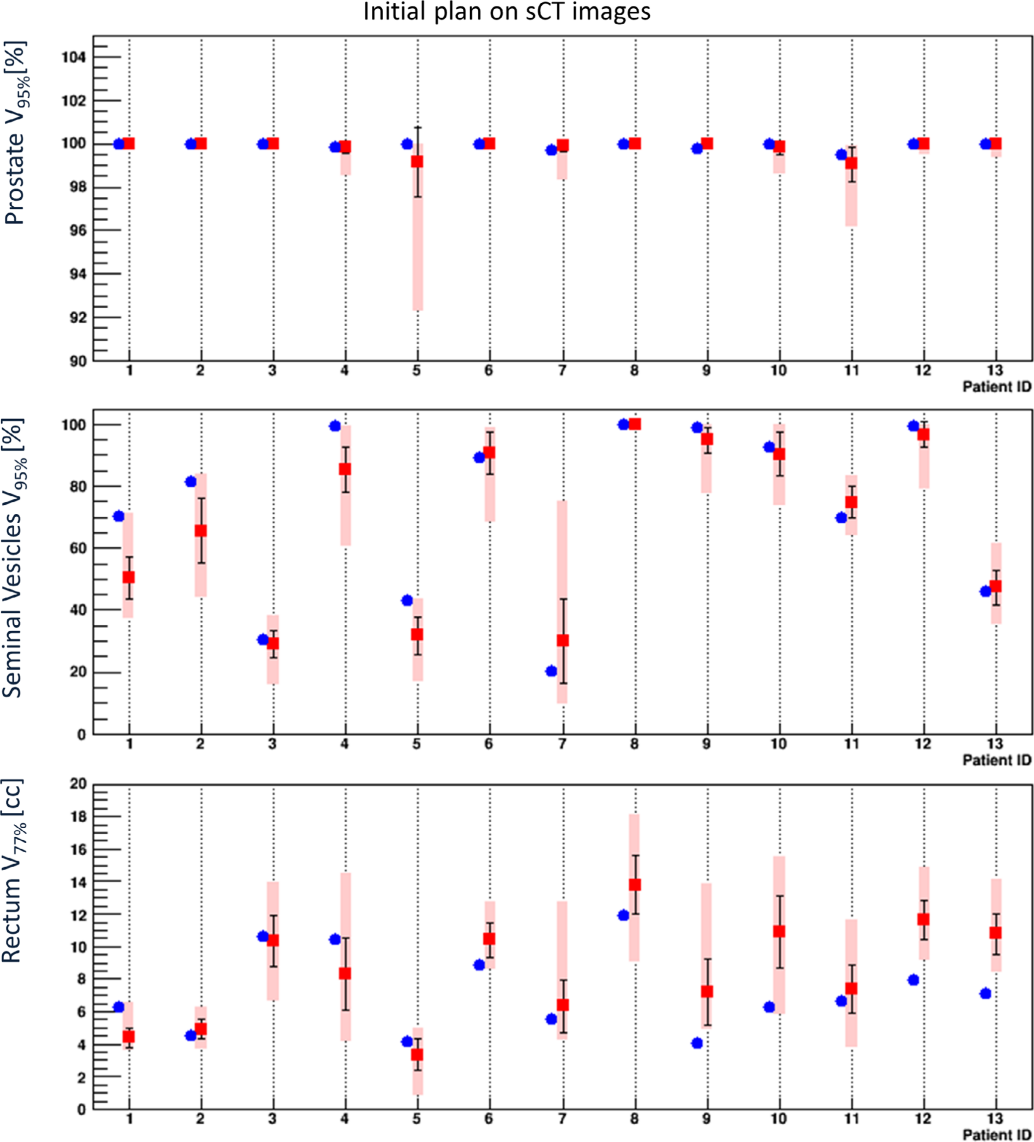
Daily dose parameters of V_95%_ for the prostate and the seminal vesicles, and the parameters of V_77%_ for the rectum evaluated based on the initial planning for all 13 patients. *Blue circles:* the initial planned values. *Red squares:* the mean values. *Error bars:* the standard deviation. *Rectangles:* minimum–maximum range among the daily treatments.

Figure 5 shows the means, standard deviations and minimum–maximum ranges for the daily dose parameters of V_95%_ of the prostate and the SVs, and the parameters of V_77%_ of the rectum, respectively. These parameters were calculated based on the initial plan using the sCT images for each patient. The dose parameters of the prostate coincided well with the original values within standard deviations for all of the patients. Nevertheless Patient #5 showed the larger standard deviation and the lower minimum value for the prostate dose parameters in comparison to others. The reason was that the larger prostate rotational variation of 8.6 degree (standard deviation) over the sagittal plane was found in this patient and almost twice larger than the variation of others. This indicates that the need for the rotational corrections to the PRB registration for soft tissue alignment for this kind of patients. This issue will be discussed later. The dose coverage of the SVs showed a large variation, and 41% of the total 348 irradiations in the higher risk treatment maintained V_95%_ values higher than the original planned value because the daily movement of SVs was large.^3^

The rectal dose parameters were almost maintained within the daily standard deviation for Patients #1–#4 and #5–#7 (in the first and second categories). However, the rectal dose parameters for Patients #8–#13 (except for Patient #11) showing rectal movement toward the anterior side tended to be greater than the original values beyond 1 SD.

**Fig. 6:**
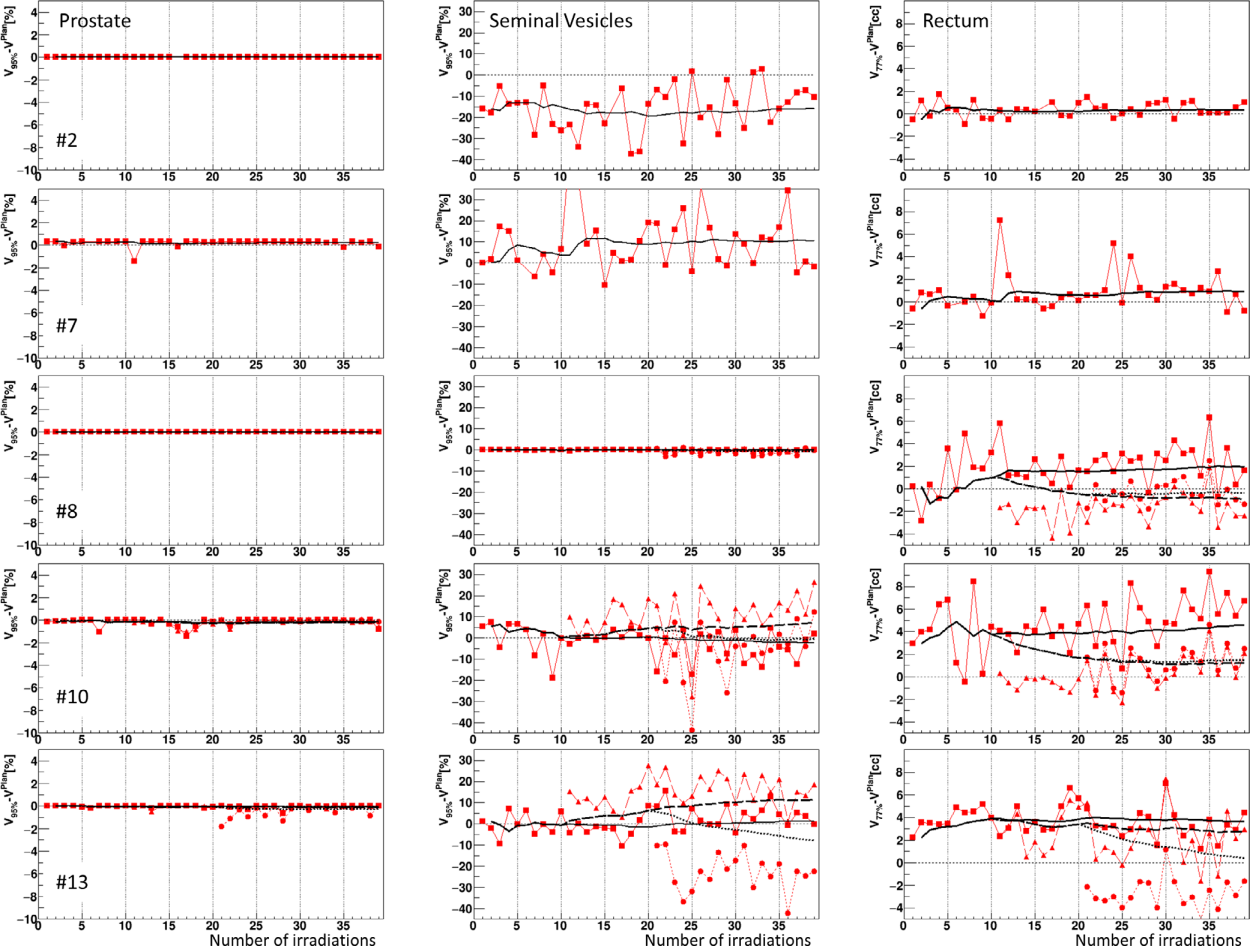
The differences (*red squares*) in the daily dose parameters from the initial planned values for V_95%_ of the prostate (*left panels*) and the seminal vesicles (*middle panels*) and V_77%_ of the rectum (*right panels*) throughout the entire treatment period for representative Patients #2, #7, #8, #10, and #13. Patient numbers are indicated in the left panels. *Solid lines:* the accumulated mean values from the first day up to the online day (indicated on the horizontal axis) for the dose parameter difference. For Patients #8, #10, and #13 categorized as the third group, the differences in the daily dose parameters from the replanning using daily CT images acquired on the 10th day (*red triangles*) and 20th day (*red circles*) are also shown. The corresponding accumulated mean values from the first day up to the online day based on the replanning using CT images on the 10th and 20th day are also shown by long dashed lines and dotted lines, respectively.

Figure 6 illustrates the differences in the daily dose parameters from the initial planned values for the prostate, the SVs and the rectum throughout the entire treatment period for five representative patients from each of the three categories. As shown in the figure, the rectal dose parameters for Patient #8 showed a tendency to increase as the irradiation numbers increased during the first 2–3 weeks of treatment and then stayed at higher values than the initial plan value, whereas the rectal dose of Patient #10 showed greater variation during the first 10 days and remained at a value that was higher than the initial plan value. On the other hand, the daily rectal dose of Patient #13 showed a positive deviation from the planned values just after the first treatment day. The size of the daily variations of the rectal dose depended on the patient.

A single or several online monitoring of daily dose parameters is sufficient to make a replanning decision for individuals such as Patients #2 and #13, whose daily rectal doses indicated a stable dose and an overdose with rather small daily random variation during the first several treatments. However, the online monitoring of daily dose parameters is not helpful for making treatment decisions due to the high randomness demonstrated by Patients #7, #8, and #10, and it may thus not be clear whether the rectal dose is going to be problematic regarding the dose distribution over the entire treatment. The high randomness can be also observed in the daily dose parameters of the SVs. For such cases, the accumulated mean dose (shown by solid lines in Fig. 6) provides the current dose condition to date, and it is much easier to estimate the trend of the dose parameters in this manner. If the tolerance level of 1.2 cc is set for a rectal overdose as the accumulated dose criterion on the 10th day of treatment, the treatments for Patients #9, #10, #12, and #13 need to be replanned. The accumulated mean dose can be used in this way to reach a primary decision for replanning on an online basis.

**Fig. 7:**
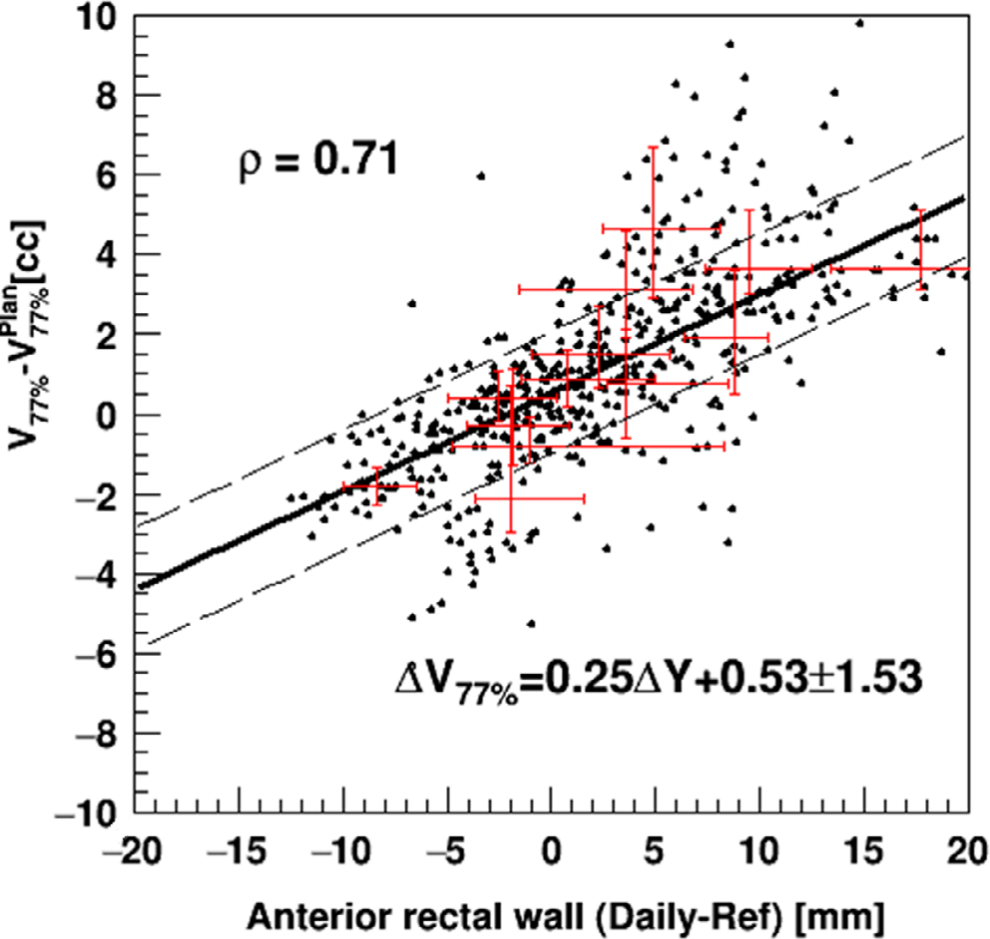
The differences in daily dose parameters of V_77%_ for the rectum from the planned value vs the daily movement of the anterior rectal wall at the tip position of the SVs based on the initial planning for all 13 patients. Plots show all data. Plots with error bars show the mean with interquartile range for each of the patients. Correlation coefficients (ρ) are also shown in the panel. *Solid and dashed lines* show the fit of all data with the linear function and the corresponding boundary of ±SD.

As an alternative approach to the evaluation of the effects of the daily movement of the rectal wall on the daily dose parameters, we plotted the deviation of the dose parameters of the rectum from the planned value as a function of the daily movement of the anterior rectal wall along the AP direction with respect to the reference, as shown in Fig. 7. The daily dose parameters showed the correlation ρ = 0.71 with the daily position of the rectal anterior wall around the SVs, and they were fitted by the linear regression function of Δ*V*_77%_ = 0.25 Δ*Y* + 0.53 with 1.53 cc of the SD of the daily dose parameters, where Δ*V*_77%_ indicates the daily dose difference from the planned value as a cubic centimeter and Δ*Y* indicates the movement of the anterior rectal wall from the reference in millimeters. This function with the standard deviation can provide the first estimate of the changes in daily dose parameters obtained by the online measurement of the positional shift of the anterior rectal wall without daily dose calculations on the dCT images.

Using this function to estimate daily rectal dose deviations for all 40 patients, the treatment of 15 patients (37.5%) including Patients #8–#13 in the third category were identified as for the replanned protocols based on the accumulated mean dose method with the tolerance of 1.2 cc on the 10th day of treatment as demonstrated in Fig. 6.

**Fig. 8:**
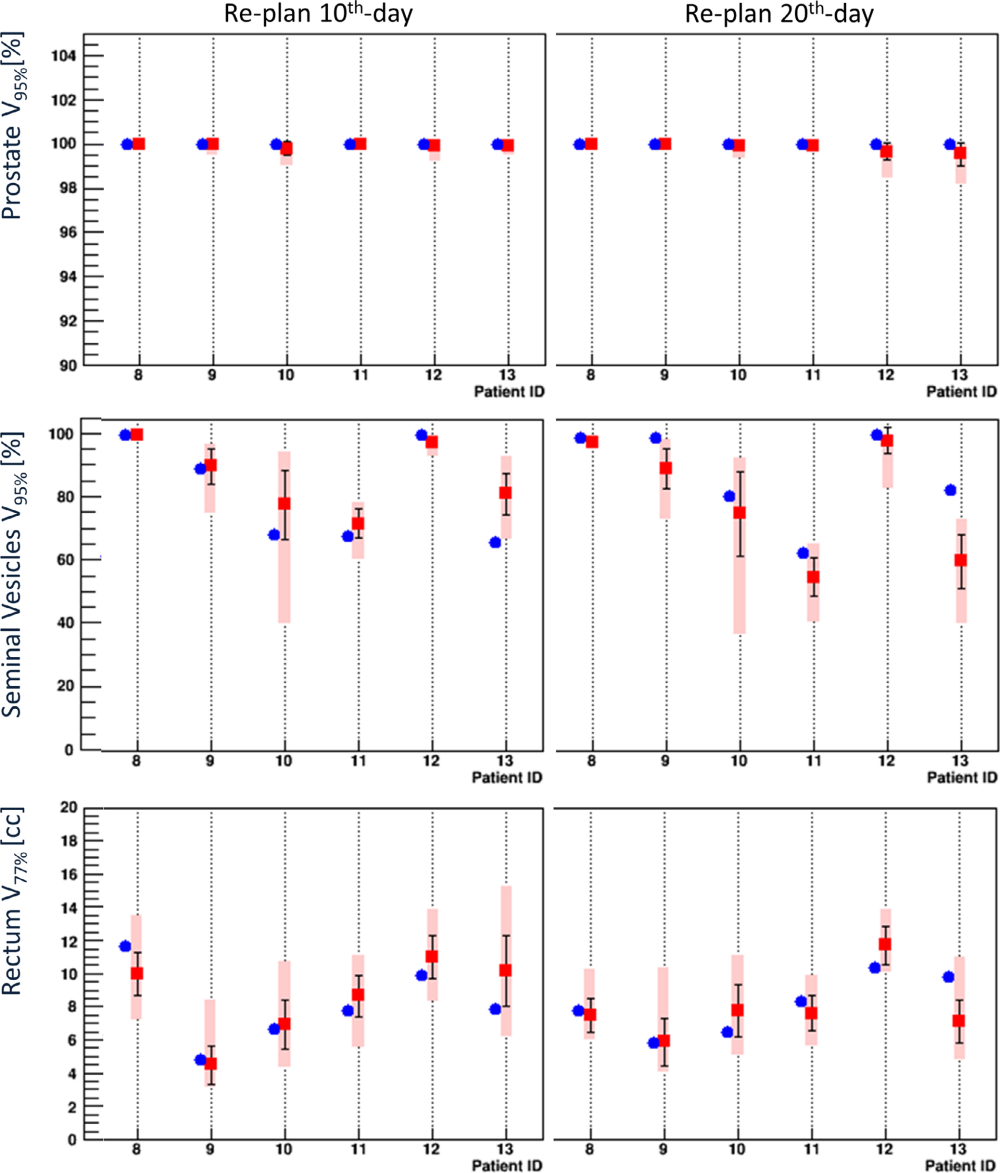
Daily dose parameters of V_95%_ for the prostate and seminal vesicles, and the parameters of V_77%_ for the rectum evaluated based on the replanning using daily CT images acquired on the 10th day (*left*) and 20th day (*right*) for Patients #8–#13 in the third category. *Blue circles:* the initial planned values. *Red squares:* mean values. *Error bars:* the standard deviation. *Rectangles:* minimum–maximum range among the daily treatments.

Figure 8 shows the daily dose parameters for Patients #8–#13 calculated by the replanning on the dCT images acquired on the 10th and 20th days. The differences in the daily dose parameters from the replanning, and the corresponding accumulated mean values from the first day up to the online day based on the replanning protocols are also shown in Fig. 6 for the representative Patient #8, #10, and #13. The daily dose parameters for the rectum tended to coincide with the planned values compared to the daily dose parameters by the original planning based on the sCT images. The replanning protocol makes it possible to maintain daily rectal doses within daily random variations over the rest of the treatment. The accumulated mean dose shown in Fig. 6 based on the replanning protocol also becomes close to the base line as the treatment day increases in comparison to the mean dose based on the initial planning. It noted that the daily dose parameters of the SVs showed large deviation among patients on the replanning too, and no systematic improvement was found on the dose parameters in the replanning protocols based on analyses of the daily rectal movement.

Table 2 summarizes the mean of V_95%_ for the prostate and the deviation of daily mean doses from the planned values for the SVs and the rectum, respectively, for each of the patient's original plans based on the sCT images and the replanning based on the dCT images on the 10th and 20th days. In table 2, the deviation of daily rectal mean doses in a percentage is also shown in addition to the one in a cubic centimeter.

**Table 2:** Mean of V_95%_ for the prostate, deviation of the mean of the seminal vesicle’s V_95%_ and rectal V_77%_ from the planned values. The values of the rectal V_77%_ in a percentage are also shown in addition to the ones in a cubic centimeter. The values inside parentheses show the standard deviation.

Ref. CT	sCT		10th dCT	20th dCT
Patient No.	1–7	8–13	8–13	8–13
Mean (Prostate, V_95%_) [%]	99.8 (0.6)	99.8 (0.4)	99.9 (0.2)	99.8 (0.3)
Mean‐plan (SV,V_95%_) [%]	−7.1 (8.5)	−0.5 (4.9)	4.7 (6.1)	−8.2 (7.7)
Mean‐plan (Rectum, V_77%_) [cc]	−0.3 (1.4)	3.0 (1.7)	0.4 (1.5)	−0.2 (1.3)
Mean‐plan (Rectum, V_77%_) [%]	−0.9 (2.7)	5.5 (3.5)	1.9 (2.8)	0.6 (2.5)

sCT: simulator CT; 10th dCT: daily CT images obtained on the 10th day of treatment; 20th dCT: daily CT images obtained on the 20th day of treatment; SV: Seminal vesicle.

Our analyses revealed that the treatment with the PRB registration was able to maintain good dose coverage of the prostate in all cases. However, the dose parameters for the patients showing the daily movement of their anterior rectal wall around the SV region toward the anterior side exceeded the averaged planned rectal dose of 7.3 ± 2.4 cc by 3.0 ± 1.7 cc, and this excess corresponds to a daily rectal movement of 9.9 ± 6.8 mm around the SVs toward the anterior side using the linear regression function shown in Fig. 7. By creating replans using the dCT images on the 10th and 20th days of treatment, the excess of the daily rectal doses could be reduced to 0.4 ± 1.5 cc and −0.2 ± 1.3 cc, respectively, and the daily dose of the rectum could be controlled to be closer to the replanning condition.

## 4 DISCUSSION

In general, the initial plan of radiotherapy treatment at our hospitals in Japan is created based on the sCT images acquired at approx. 10 days before the patient's first treatment. Then, during our in‐room CT image‐guided proton treatment for prostate cancer, the daily volume of the bladder is monitored by ultrasound scans and, when the bladder is not sufficiently distended, the patient is given water to drink. In addition, the defecation and exhaust gases are managed in order to maintain the condition of the rectum so that it matches that observed on the sCT images. Despite this pretreatment, as shown in Fig. 2, the means of the daily movement over the SI position showed a tendency in which the anterior rectal wall at the superior region moves toward the anterior direction compared to the movement on the sCT images. It can be expected that the shift of the rectal wall to the anterior direction causes an excess of the planned dose in the accumulated daily dose of the rectum.

By referring to dCT images as the days pass, it can be observed that the mean of the rectal wall tends to coincide with the baseline. The change of the means between sCT images and dCT images taken on the first day is the most significant and the deviation of the mean around the SV region decreases exponentially over a span of 3 days. This may be because patients gain familiarity with the preparations for CT scanning as the days pass and become better able to replicate the muscle tension around the buttocks and thighs while lying on the couch during treatment. In addition, patients become used to maintain greater stability of the rectal shape during defecation and exhaust gas examinations.

The present study's daily dose calculations based on the sCT images with the PRB registration revealed that, although the dose coverage of the prostate in almost all of the irradiations satisfied the condition of V_95%_ ≈ 100%, the mean of the daily rectal dose exceeded the original planned value for the patients who showed rectal movement around the SV region toward the anterior direction, as mentioned earlier. For such patients, our data showed that the replanning with the reference CT after 10 days had passed was effective to maintain the rectal dose constraint for the rest of the patient's treatment. This result demonstrated that, in the prostate registration using fiducial markers (which is considered the standard approach for image guidance in proton therapy), the daily rectal dose might exceed the original planned conditions due to the rectal movement and deformation around the SV region toward the anterior direction. Since clinicians cannot identify the location of soft tissues via image registration using fiducial markers in combination with conventional kV x‐ray imaging, the use of CT image guidance could help address this problem by monitoring daily changes of the rectal shape and its dose parameters.

We propose an accumulated mean dose method to follow the daily dose changes. This method can be used to make a primary judgment regarding replanning on an online basis during the early period of each patient's treatment with CT image guidance. In particular, this method used during the treatment can be useful to monitor daily dose parameters for patients whose daily random variation of the anterior rectal wall is rather large and results in a rectal overdose. Our data showed that the accumulated mean dose up to 10th day of treatment and the mean dose over the whole treatment showed the linear correlation ρ=0.97 with the residual error of 0.6 cc (RMS). Assuming Gaussian distribution on the residual, if we consider 2.5 cc as a tolerance level for the mean of the rectal overdose during whole treatment, the probability to identify these treatment on the 10th day by setting 1.2 cc tolerance threshold on the accumulated mean dose was estimated to be 97.7% due to more than two sigma separation, that is, 2.5 cc > 1.2 cc + 2 × 0.6 cc. Nevertheless, since the tolerance value was estimated using our limited data, we recommend that the value need to be validated before clinical application.

In combination with this method, a dose verification system dedicated to CT image guidance is desired to achieve more precise approaches to the monitoring and validation of the changes in daily dose parameters with considerations of the online image registration just prior to the patients' treatment. For this system, precise automatic contouring and deformed image registration using deep neutral networks may be necessary in order to accumulate daily dose distributions on the reference images.^11^ To our knowledge, such a system is not yet clinically available at the proton therapy facilities equipped with CT image guidance, and we are currently researching this issue.

As an empirical and alternative approach, we also provided linear regression functions based on our data sets to estimate the daily dose deviation of the rectal V_77%_ with the knowledge of the daily shift of the anterior rectum. Although the precision for the estimation of the rectal dose parameters is limited at 1.5 cc, this approach does not require dose calculations or anatomical contouring, and thus it can be easily incorporated into clinical workflows.

The beam scanning method has been widely adopted as a three‐dimensional irradiation technique in particle therapy facilities, and in principle, online adaptive replanning is possible based on this scanning method. However, technical problems remain to be resolved, that is, automatic segmentations, first planning optimization, and planning quality assurance. We propose (a) the use of an optimal period for replanning to spare the rectal dose by analyzing the pelvic anatomical movement on daily CT images acquired throughout prostate treatment, and (b) an approach to identify problematic patients for the daily rectal dose during the first period of the patient's treatment. The results of our analyses demonstrate that the replanning protocol conducted during the treatment after 10 days have passed was effective to control the target dose coverage as well as the rectal dose, and the proposed protocol is clinically applicable for prostate treatment using the scanning method as well as the passive method. However, if the daily rectal dose variation before the replanning period is rather large, higher doses may accumulate in the rectum during this period for particular patients. In such cases, stronger dose constraints of the rectum compared to the conventional protocol in replanning must be set to compensate for the accumulated rectal dose over the whole treatment, and for some patients it will be necessary to compensate for the maintenance of the rectal dose constraint due to the dose coverage of the target.

In order to avoid the above situations, dose distributions on daily CT images should be validated more frequently during the first period of the treatment, and the frequency of the replanning needs to be increased. Since the changes in the mean values of the prostate and the anterior rectal wall between the simulator CT images and the first daily CT images were observed to be the most significant, the replanning based on daily CT images during the first week of treatment can be considered to be effective for this issue and is a clinically realistic approach, particularly for treatment using a beam scanning system with in‐room CT image guidance.

The applications of the rotational correction (pitch angle) over the sagittal plan in addition to two transversal corrections performed in the PRB registration for the soft tissue alignment may improve the prostate dose coverage and also reduce the daily positional deviation of the rectal anterior side around the SV region as well as SVs, which may result in sparing the daily rectal dose further. The daily random error of the prostate rotation over the sagittal plane was found to be significantly large in order of 7–10 degree.^12^ Our data showed the random error of 2–9 degree. Generally the rotational movement of the patient couch over the sagittal plane is limited mechanically, and the limitation is within 2 degree in the case of our proton facility. Therefore this issue has to be considered to develop the registration method taking the prostate rotational variations into account. Moreover such registration method for the soft tissue alignment can be realized in the recent proton therapy facility with the 6 degree‐of‐freedom robotic couch in combination with CT‐image guidance,^1^ and we will study the effectiveness of the proposed replanning protocol based on the PRB registration method including rotational angle corrections in the future.

Our data showed that the daily dose coverage of the SVs varied among patients, and the replanning protocols based on the rectal movement could not improve the maintenance of the daily parameters as planned. This was because the daily movement of the tip portion of the SVs located at each of left and right side of the prostate was found to be larger than the movement at the center of the prostate,^3^ and may have no correlation with the daily movement of the anterior rectal wall. In addition, the field margin around the tip portion of the SVs used for opposed lateral proton beams was not sufficient to keep the dose coverage due to the rectal dose contain. Adaptive therapy by optimizing the aperture shape of the multileaf collimator or the proton range adjustment for anterior‐oblique beams may overcome this issue in combination with in‐room CT image guidance.^13^

The use of hydrogel rectal spacers for proton prostate treatment is considered to be effective to reduce the rectal dose, resulting in lower toxicity by making space between the rectum and prostate. The use of this technique can be expected to increase, but the stability of implantation as well as the absorption rate of a biodegradable compound over the treatment period must be studied by using several imaging modalities to adjust treatment plans appropriately.^14^, ^15^ In particular, the implantation stability around the SV region is questionable, since the daily movement of the rectum around this region was found to be significant herein. However, this topic is beyond the scope of this paper and our database, and it will be examined elsewhere.

The limitations of this work are as follows. Since our findings were based on image datasets acquired only at our single facility for a limited number of patients, the stability of the rectal shape at other proton facilities might show characteristics that differ from our datasets even if the same immobilization method and pretreatment are used. We thus recommend that the position of the rectum as well as the prostate during treatment be monitored with CT image guidance, and the influence of anatomy changes on daily dose parameters should be investigated by using an approach that is similar to what we have described before the clinical application of the protocol that we have described.

## 5 CONCLUSION

We analyzed 1483 sets of daily CT images acquired throughout the proton therapy for 40 patients, which is a fourfold greater number of image sets than used in our previous study. The daily rectal movement was measured along the AP direction with respect to the reference CT images (including daily acquired images) by simulating the PRB registration. The results of our analyses demonstrated that the daily movement of the rectal anterior side around the seminal vesicle region tends to move toward the anterior side compared to that in the simulator CT images, resulting in a higher rectal dose in daily treatment, and the mean of the daily movement gradually decreases as the days pass. Our results also demonstrated that the accumulated mean rectal dose during the treatment can be useful to make a primary online decision regarding replanning for a patient with large daily variation of the anterior rectal wall movement that results in a rectal overdose. For such cases, we observed that replanning with the reference CT image after 10 days have passed was effective to spare the rectal dose.

## CONFLICT OF INTEREST

None of the authors have any financial interest or personal relationships with other persons or organizations that could inappropriately influence our work.
